# NMLPA: Uncovering Overlapping Communities in Attributed Networks via a Multi-Label Propagation Approach †

**DOI:** 10.3390/s19020260

**Published:** 2019-01-10

**Authors:** Bingyang Huang, Chaokun Wang, Binbin Wang

**Affiliations:** School of Software, Tsinghua University, Beijing 100084, China; hby17@mails.tsinghua.edu.cn (B.H.); wbb18@mails.tsinghua.edu.cn (B.W.)

**Keywords:** overlapping community detection, attributed networks, multi-label propagation, node similarity

## Abstract

With the enrichment of the entity information in the real world, many networks with attributed nodes are proposed and studied widely. Community detection in these attributed networks is an essential task that aims to find groups where the intra-nodes are much more densely connected than the inter-nodes. However, many existing community detection methods in attributed networks do not distinguish overlapping communities from non-overlapping communities when designing algorithms. In this paper, we propose a novel and accurate algorithm called Node-similarity-based Multi-Label Propagation Algorithm (NMLPA) for detecting overlapping communities in attributed networks. NMLPA first calculates the similarity between nodes and then propagates multiple labels based on the network structure and the node similarity. Moreover, NMLPA uses a pruning strategy to keep the number of labels per node within a suitable range. Extensive experiments conducted on both synthetic and real-world networks show that our new method significantly outperforms state-of-the-art methods.

## 1. Introduction

With the rapid development of real-world networks such as social networks and sensor networks [1,2,3,4], network analysis has become a hot issue nowadays [5,6]. Community detection, where a community is conventionally defined as a subgraph with dense internal connections and sparse external connections, is an essential task for the network analysis. For example, in social networks, community detection allows users to find groups of friends who have the same or similar hobbies. In protein-protein interaction networks, communities correspond to functional modules of interacting proteins [7]. Moreover, community detection can be applied in many other network analysis tasks, such as edge prediction, missing attributes inference [8,9] and community search [10,11].

**Figure 1:**
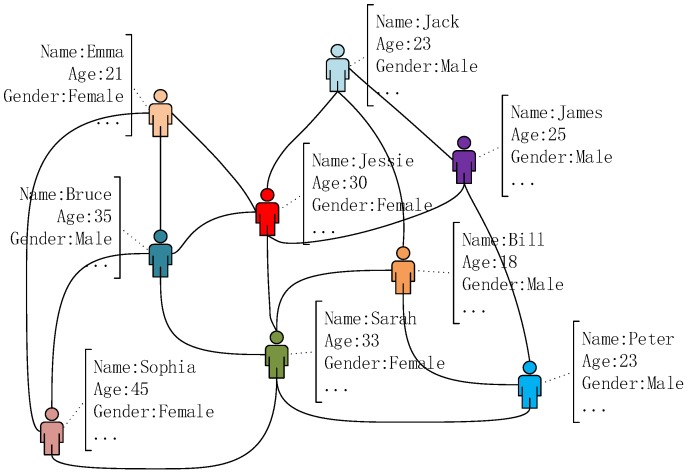
A social network with users as nodes. Texts next to the nodes are the profiles of the users, which can be regarded as node attributes.

One of common application scenarios of community detection is the attributed network where nodes tend to have attributes. For example, papers in citation networks have some related keywords as their attribute information; in social networks, the profiles of users can be regarded as node attributes (Figure 1); and, in sensor networks, the power, congestion and other resources available in the network nodes provide attribute information for sensor nodes [12]. Community detection in attributed networks not only needs to explore the network topology, but also makes full use of the node attributes [13,14,15]. Node attribute information is a crucial point for community detection in attributed networks since these attributes are helpful to decide the community memberships for those nodes with few links. In this context, many remarkable studies about community detection, which only consider the structure information of the network, are not suitable for attributed networks. For example, many label propagation based methods [16,17] have this limitation. Since label propagation based methods need to attach one or more labels to each node of the network when label propagating, it is technically challenging to explore node attributes in the label propagation process. As a result, plenty of label propagation based methods, which ignore node attributes but achieve high performances in general networks, cannot be simply applied to attributed networks.

In general, communities can be categorized as overlapping communities and non-overlapping communities. However, most existing attributed community detection algorithms, in which both network structures and node attributes are developed, only can handle the non-overlapping community detection problem [13,18,19,20,21] or just treat both types of communities equally [22,23]. The latter algorithms only distinguish overlapping and non-overlapping communities in the final community output stage, which may result in unsatisfactory results in overlapping community detection. Therefore, several overlapping community methods in attributed networks have been proposed to specially solve this problem [24,25,26]. However, the applicability of these methods is limited, as they are only suitable for certain types of networks or very time-consuming for large attributed networks. For instance, the Circles method [24] is only able to handle ego networks, where a user’s ego network in social networks is defined as connections between her friends, and needs ten hours to process an attributed network with 5000 nodes [27].

In this paper, we propose and develop a novel and high-accuracy overlapping community detection algorithm in attributed networks, called Node-similarity-based Multi-Label Propagation Algorithm (NMLPA), to address the problem of applying label propagation to the attributed network analysis. NMLPA integrates the topology information and node attributes of the network during the label propagation process. By working out the similarities between nodes from the node attribute information, NMLPA is able to conveniently leverage the node attributes in the label propagation process. NMLPA also uses multiple labels instead of a single label for one node to make communities overlapping, i.e., one node can belong to multiple communities. Moreover, a pruning strategy is used in NMLPA to adjust the number of labels per node, which makes the final community number within a suitable and accurate range.

Our main contributions are summarized as follows.

- We propose a novel overlapping community detection algorithm, which considers both the network structure and node attribute information, to identify underlying community structures in attributed networks.
- We creatively combine the node attribute information with the multi-label propagation process in the way of mapping the node similarity to the edge weight and formulate our Node-similarity-based Multi-Label Propagation Algorithm (NMLPA) to uncover overlapping communities in attributed networks. To the best of our knowledge, NMLPA is the first overlapping community detection method that leverages the node attribute information in the multi-label propagation process. Furthermore, NMLPA uses a pruning strategy to control the number of labels per node when label propagating, which leads to an appropriate range of the final community number.
- We carefully design overlapping community detection experiments in both synthetic networks and real-world networks. Results of these extensive experiments show that our proposed NMLPA significantly outperforms the state-of-the-art methods.

## 2. Related Work

Works related to this paper include community detection in attributed networks and label propagation based community detection.

### 2.1. Community Detection in Attributed Networks

Community detection algorithms in attributed networks are widely studied in recent years. There are several classes about community detection methods in attributed networks such as topic-model-based methods, NMF (Nonnegative Matrix Factorization)-based methods, clique-based methods and social circles methods.

Topic-model-based methods [8,28,29] generally use topic models to analyze the attributed networks. Modeling by generating community structures from node attributes and network structures, these methods can make full use of the strength of topic models. Therefore, these methods achieve nice performance in some types of attributed networks. However, the assumption of soft community memberships in these works makes them unrealistic in terms of overlapping communities [30].

NMF-based methods [22,23] utilize the technique of NMF to combine the network structure and node attributes. Since these methods need lots of time to accomplish the factorization process, they are not suitable for large attributed networks.

Clique-based methods [31,32] model the attributed networks based on the assumption that the given network can be divided into subgraphs of clique patterns. These works find communities by joining the paradigms of subspace clustering and dense subgraph mining, which require nodes have high similarity and are densely connected in the same community, respectively.

Social circle methods [24,33] aim to discover communities in attributed social networks. In these works, communities are defined as the social circles of the user-specific ego networks. Since these methods are also time-consuming [27], they are only able to handle some relatively specific networks.

### 2.2. Label Propagation Based Community Detection

Label propagation is a hot research topic in the field of community detection, in which each node propagates the label to its neighbor nodes by the edges between them. Label Propagation Algorithm (LPA) [16] was proposed to find the non-overlapping communities in near linear time. In each iteration of this algorithm, each node is assigned a label which has the maximum number with respect to the labels of its neighbors. This algorithm can be completed in linear time because each iteration only takes O(m) time, where *m* is the number of edges.

To solve the problem of overlapping community detection, several extended methods based on LPA [16] are proposed in recent years. Community Overlap Propagation Algorithm (COPRA) [34] attaches a set of pairs (*c*, *b*) to each vertex, where *c* is a community identifier and *b* is a belonging coefficient. Balanced Multi-Label Propagation Algorithm (BMLPA) [35] requires that community identifiers of one vertex should have balanced belonging coefficients, which allows nodes to belong to any number of communities without a global limit on the largest number required by COPRA. Speaker-listener Label Propagation Algorithm (SLPA) [17] uses a speaker-listener interaction dynamic process to ensure that each node can own multiple labels. Weighted Label Propagation Algorithm (WLPA) [36] extends SLPA by introducing a similarity between any two nodes based on the labels each node has received during the label propagation. However, these label-propagation-based methods are not able to handle attributed networks since all of them exclude the node attribute information in the label propagation process. Different from these existing label propagation methods, our work makes full use of the attribute information of nodes, thus it can be well applied to attributed networks.

Furthermore, referring to works about the analysis of more complex networks such as heterogeneous networks [37] and multiple networks [38,39], our method could be extended appropriately and then applied to more complex networks.

## 3. Problem Definition

Let G=(V,E,L,T) be a directed but unweighted attributed network. $V=\{v_{1},v_{2},\ldots ,v_{n}\}$ is the set of *n* nodes, E⊆V×V is the set of directed edges, $L=\{l_{1},l_{2},\ldots ,l_{s}\}$ is the set of node attributes, and $T\in {\left\{0,1\right\}}_{n\times s}$ is the node attribute matrix where the *i*-th row, denoted by an *s*-dimensional binary-value vector, represents the attributes of node $v_{i}$. Please note that, in the rest of this paper, our discussions focus on directed attributed networks, but our method can be easily extended to handle undirected attributed networks. The problem of overlapping community detection is to partition the network *G* into *k* communities $C=\{c_{1},c_{2},\ldots ,c_{k}\}$ that satisfy the following conditions:

1. In terms of network structure, the nodes in the same community are densely connected while the nodes in different communities are sparsely connected.
2. In terms of node attributes, the nodes in the same community tend to have similar attribute values while the nodes in different communities tend to have diverse attribute values.
3. $c_{i}\cap c_{j}\neq \emptyset$ is allowed to exist.

**Figure 2:**
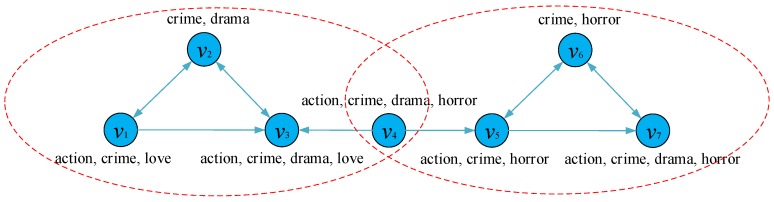
An example of community detection in an attributed network. The red dotted lines represent the boundaries of the communities. Texts next to nodes represent their node attributes.

For example, the two communities shown in Figure 2 may be a nice partition of the attributed network since these communities meet the above conditions very well.

## 4. Node Similarity Based Multi-Label Propagation

In this section, the NMLPA algorithm is proposed. At first, the overview of NMLPA is introduced. Then, the main components of NMLPA are presented in detail. Next, the complexity of NMLPA is analyzed to show its efficiency. Finally, the parameter settings are brought forward.

### 4.1. Overview

As for any community detection algorithm in attributed networks, the key issue is how to comprehensively utilize both the topology information of the network and the attribute information of nodes. NMLPA skillfully maps the node attribute information to the weights of edges. Therefore, our algorithm is capable of using the attribute information of nodes in the label propagation process easily. Since label propagation methods have been proved to make full use of the network topology information when labels propagate by edges [16,35], NMLPA is probably a great solution for overlapping community detection in attributed networks.

### 4.2. The NMLPA Algorithm

The NMLPA algorithm includes two main steps: the procedure for weighted network construction and that for multi-label propagation. The weighted network construction takes an unweighted attributed network *G* as the input and constructs a new weighted network $G^{{}^{\prime }}$ without node attributes based on the attribute information of nodes in *G*. The multi-label propagation procedure, running on $G^{{}^{\prime }}$, propagates multiple labels among nodes according to the network topology information and the weights of the edges. When the process of the multi-label propagation ends, we can infer the communities of the attributed network from the labels attached to each node.

Algorithm 1 shows the procedure of our approach. We first construct a weighted network $G^{{}^{\prime }}$ from the input network *G* on Line 1. In the label propagation process of Lines 3–5, we iterate *t* times over the weighted network after an initialization of labels at all nodes on Line 2. For each iteration *i*, we use the multi-label propagation method to update the labels of all nodes based on the labels at iteration i−1. When this main loop ends, the final communities *C* can be calculated and returned on Lines 6–7 after a post processing stage of the labels at iteration *t*.

**Algorithm 1:** The NMLPA algorithm.

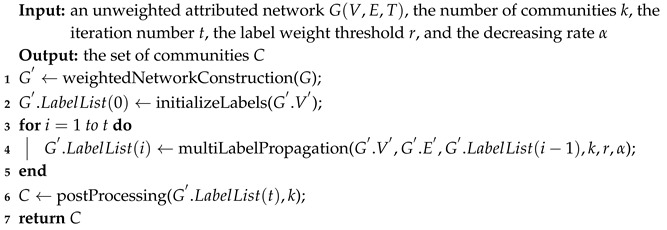



#### 4.2.1. Weighted Network Construction

To make full use of the information of node attributes, we construct a weighted network based on the node similarity of the original unweighted attributed network. It maps the similarity between two nodes into the weight of the edge between them using a similarity evaluation metric. In this paper, we define the similarity between node $v_{i}$ and node $v_{j}$ as the following:

(1) $$Similarity\left(v_{i},v_{j}\right)=1+\sum_{k=1}^{s}I_{\left\{2\right\}}\left(T_{ik}+T_{jk}\right)$$

where $I_{\left\{2\right\}}\left(T_{ik}+T_{jk}\right)$ is the indicator function, which gives 1 when $T_{ik}+T_{jk}$ is equal to 2 and gives 0 otherwise. This value calculates the number of common attributes between node $v_{i}$ and node $v_{j}$ plus 1. We design such a formula so that the weight of any edge is not equal to zero.

**Figure 3:**
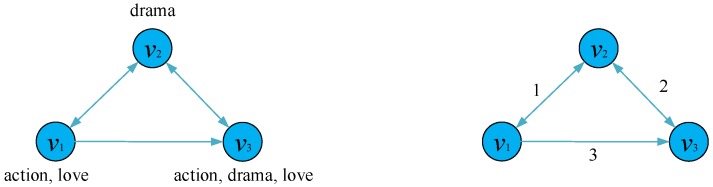
An example of the reconstruction of the weighted network $G^{{}^{\prime }}$ from the unweighted attributed network *G* based on the node similarity. Texts next to nodes represent their node attributes.

Algorithm 2 shows the details of the weighted network construction. We first copy the node set of *G* to $G^{{}^{\prime }}$ on Line 2. Since each edge of $G^{{}^{\prime }}$ is a triad ($v_{i},v_{j},weight\left(v_{i},v_{j}\right)$) whose last element represents the weight between $v_{i}$ and $v_{j}$, we need to calculate the similarity between any two nodes which have an edge in *G* according to Equation (1) on Lines 3–8. An example that explicates this algorithm is shown in Figure 3, in which the weighted network $G^{{}^{\prime }}$ is distinctly more concise and easier to use.

**Algorithm 2:** Weighted network construction (*G*).

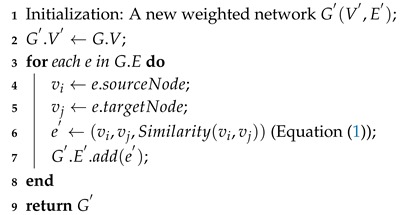



#### 4.2.2. Multi-Label Propagation

Two data structures are used during the process of the multi-label propagation. For each node of $G^{{}^{\prime }}$, our approach maintains an ordered label list with the order representing the weight of the corresponding label. Since this label list excludes the weight of the corresponding label, we need a label dictionary, i.e. a temporary variable of the dictionary type, to store the weights of labels of each node in each iteration of the multi-label propagation. Before the multi-label propagation starts, an initialization of the label list for each node in $G^{{}^{\prime }}$ is done by assigning the node id as the only element in the label list.

Algorithm 3 shows the main process of the multi-label propagation. Our method first iterates all edges in $G^{{}^{\prime }}$ to propagate the labels generated in the last iteration. For each edge, we propagate all labels in the label list of the source node to the target node in a sequence of decreasing weights, as shown on Lines 4–18. In this stage, the label weight decreases by α, which is a small number far less than 1.0, from 1.0 to the minimum positive decimal it can achieve. To make full use of the edge weights calculated in Algorithm 2, the product of the edge weight and the label weight is used as the final label weight, as shown on Lines 10 and 12.

To avoid generating too many labels in one iteration, a pruning operation is executed on Lines 20–24 after the multi-label propagation finishes. For the label dictionary of each node in $G^{{}^{\prime }}$, we remove the labels with the weights less than the threshold *r* and then select the top-*k* labels with the highest weights in order to make up the final label list, in which *k* is the community number of *G*.

**Figure 4:**
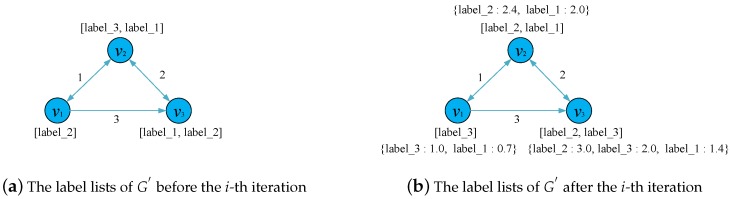
An example of an iteration in the multi-label propagation algorithm with *k* = 2, *r* = 1.0 and *α* = 0.3. Texts next to nodes represent the label list (enclosed in brackets) and the label dictionary (enclosed in braces) of the node.

The example in Figure 4 shows the details of the multi-label propagation and the pruning operation in one iteration. It is worth noting that, at each iteration of the multi-label propagation process, the synchronous mode [16] is used to update the labels of each node. That is to say, what the target node of any edge receives at iteration *i* is the labels of the corresponding source node at iteration i−1. Therefore, the update order of the edges does not matter in the process of the multi-label propagation.

**Algorithm 3:** Multi label propagation (V,E,labelList,k,r,α).

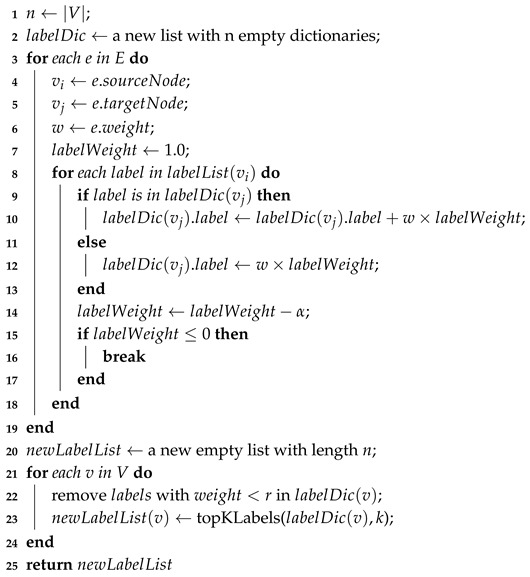



#### 4.2.3. Post-Processing

The detection of overlapping communities is performed when the label information is post-processed. Given the label lists of all nodes in $G^{{}^{\prime }}$, NMLPA counts the number of nodes each label belongs to and then selects the top-*k* labels with the largest number of nodes as the final communities. Besides, to make the final communities cover all nodes in *G*, we add all nodes that are not in the final communities to the largest community. It is noteworthy that the number of the final communities may be less than *k* since the label lists generated by the multi-label propagation may have insufficient different labels.

### 4.3. Complexity Analysis

The time complexity of each step of NMLPA (Algorithm 1) is estimated below. In the following discussion, *n* represents the number of nodes, *m* represents the number of edge and *s* is the number of node attributes. *k*, *t*, *r* and α are the parameters described in Algorithm 1.

1. On Line 1 of Algorithm 1, the construction of the weighted network $G^{{}^{\prime }}$, shown in Algorithm 2, is divided into two parts: the construction of the node set and the construction of the edge set, which take O(n) and O(ms) time respectively. Thus, the total time is O(n+ms).
2. The initialization of label list takes time O(n) on Line 2 of Algorithm 1.
3. On Lines 3–5 of Algorithm 1, each iteration of the multi-label propagation, shown in Algorithm 3, first takes O(n) time to initialize the label dictionary. For each edge in $G^{{}^{\prime }}$, one iteration of the multi-label propagation takes O(l) time to propagate labels from one node to another, where *l* is minimum of *k* and $\left\lceil \frac{1}{\alpha }\right\rceil$. The pruning operation in one iteration needs O(nk) to initialize the new label list and O(mllgml) time to execute the pruning operation since the total number of repeatable labels is not greater than ml and here we use the quick sorting to select the top-*k* labels for each node. Therefore, the total time of the multi-label propagation process is O(t(nk+mllgml)).
4. In the stage of post-processing on Line 6 of Algorithm 1, using a hash table, our method counts the number of nodes each label belongs to in O(nk) time since the number of labels for each node is not greater than *k*. Because the number of distinguishing label is not great than the number of nodes, we need O(nlgn) time to select the top-*k* labels and O(n) time to make communities cover all nodes. Thus, the total time of this stage is O(nk+nlgn).

To sum up, the total time of our approach is O(ms+tmllgml+tnk+nlgn) after some necessary simplifying operations. For a non-sparse network, where *n* is less than *m*, the total time is O(ms+tmllgml+tnk). Furthermore, if we assume *s* is small enough, NMLPA is performed only in O(tmllgml+tnk) time.

### 4.4. Parameter Settings

The parameters of NMLPA, which include the community number *k*, the iteration number *t*, the label weight threshold *r* and the decreasing rate α, are set in this part. As in many existing community detection algorithms, we assume that the community number *k* is the given number of the ground-truth communities [22,23]. The iteration number *t* controls the running time of our method since the label propagation stops when the number of iteration reaches *t*. Under the premise of collecting sufficient label information, we set t=10 to make the running time of our method in an acceptable range. We also fix r=3 in all experiments of this paper.

As for the decreasing rate α, it is obvious that this parameter is related to the degrees of nodes and the size of the communities. In detail, the average degree denoted by AD, the average size denoted by AS and the average community memberships denoted by AN (see Table 1) could be used to determine the value of α as following:

(2) $$\frac{1}{\alpha }=k_{1}AD+k_{2}AS+k_{3}AN+b$$

where $k_{1}$, $k_{2}$, $k_{3}$ and *b* are the related coefficients predefined in advance. In this work, we empirically set $k_{1}=10$, $k_{2}=-21$, $k_{3}=98$ and b=−75. Since α could be under zero according to Equation (2), we simply set α equal to $\frac{1}{AN}$ when the value calculated by Equation (2) is negative.

## 5. Experiments

Experiments on both synthetic networks and real-world networks were performed to evaluate the effectiveness of our proposed NMLPA. We also performed parameter sensitivity analysis and detected communities analysis of our algorithm. Our algorithm was implemented in Python 2.7, and all experiments were conducted under Ubuntu 16.04 with a 10-core Intel Xeon E5-2630 CPU (2.20 GHz) and 320 GB main memory except for the experiments of SCI [22] and CDE [23], which were conducted under Windows Server 2008 R2 with two 8-core Intel Xeon E5-2650 CPUs (2.00 GHz) and 256 GB main memory.

### 5.1. Evaluation on Synthetic Networks

We first evaluated NMLPA on synthetic networks constructed using the extension of LFA benchmark [40], which allowed us to generate a directed network with overlapping communities. In our experiments, we set the network with size n=5000. The average degree *k* and the maximum degree maxk of all nodes were 10 and 50, which were at the same order as most real-world networks. The minus exponents of the node degree and community size, which are governed by the power laws, were set to 2 and 1. Other configuration parameters about the community were set as follows: the community size varied from 20 to 100; and the number of overlapping nodes, represented by $O_{n}$, was set to be 40% of the total number of nodes. We chose the mixing parameter μ, which is the expected fraction of the cross-community edges, and the number of community memberships of the overlapping nodes represented by $O_{m}$ as independent variables to generate multiple synthetic networks. In detail, to create harder community detection tasks, we varied μ from 0.1 to 0.3 and increased the value of $O_{m}$ from 10 to 20. These settings helped us construct dozens of networks with ground-truth communities for testing.

Since our algorithm takes an attributed network as the input, we needed to generate some attributes for each node in these synthetic networks. We drew lessons from another paper [22] to generate the attributes of each node as follows. For each node in the network with *k* communities, we generated a *k*-dimensional binary attribute list based on the community memberships of this node. Concretely, for the *i*-th attribute of some node *v*, we used a binomial distribution with mean $\rho_{in}$ to generate the value of this attribute if *v* belongs to *i*-th community. If not, we used a binomial distribution with mean $\rho_{out}$ to generate the attribute value. Totally, we generated multiple attributes, which are closely related to the community structures, for each node in these synthetic networks. It is obvious that the value of $\rho_{in}$ should be greater than $\rho_{out}$ and the larger difference between these two parameters represents the better distribution of the node attributes. In this work, we set $\rho_{in}=0.8$ and $\rho_{out}=0.2$, respectively.

As mentioned above, our algorithm makes full use of the attribute information in the multiple label propagation process. The best method to see how well it utilizes the attribute information in the label propagation was to compare the performance between NMLPA and NMLPA_NonAttr, in which we treated the weight of each edge as value 1. For an intuitive representation, we call the former schema as NMLPA_Attr in the following experiments on synthetic networks. We also chose SLPA [17] as our baseline method, since it also uncovers overlapping communities by means of the label propagation and achieves good performance in community detection tasks by only using the information of the network structure.

To evaluate the performances of these community detection methods, we adopted F1-score and Jaccard Similarity, which are also used in other community detection methods [41], as our evaluation metrics. These two metrics try to match every detected community with the most similar ground-truth community and match every ground-truth community with the most similar detected community. Formally, given a set of ground-truth communities $C^{*}$ and a set of detected communities *C*, the evaluation functions of F1-score and Jaccard Similarity are defined as follows:

(3) $$\frac{1}{2|C^{*}|}\sum_{C_{i}^{*}\in C^{*}}^{}\max_{C_{j}\in C}\delta \left(C_{i}^{*},C_{j}\right)+\frac{1}{2|C|}\sum_{C_{j}\in C}^{}\max_{C_{i}^{*}\in C^{*}}\delta \left(C_{i}^{*},C_{j}\right)$$

where $\delta (C_{i}^{*},C_{j})$ is a similarity measure between two community sets. In the F1-score metric, this similarity measure is defined as the harmonic mean of $C_{i}^{*}$ and $C_{j}$ while, in the Jaccard Similarity metric, $\delta \left(C_{i}^{*},C_{j}\right)=\frac{|C_{i}^{*}\cap C_{j}|}{|C_{i}^{*}\cup C_{j}|}$. Both metrics are between 0 and 1 and represent better results with larger values.

**Figure 5:**
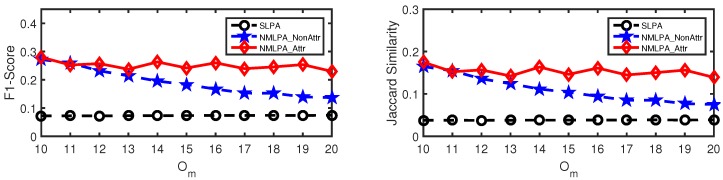
Performance comparison (in terms of F1-score and Jaccard Similarity, respectively) on synthetic networks with μ=0.1.

**Figure 6:**
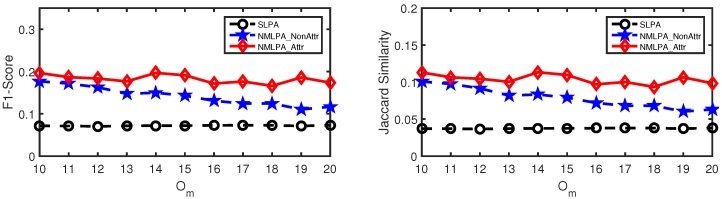
Performance comparison (in terms of F1-score and Jaccard Similarity, respectively) on synthetic networks with μ=0.3.

The results of community detection methods in synthetic networks are presented in Figure 5 and Figure 6. They show that the NMLPA methods, including NMLPA_NonAttr and NMLPA_Attr, significantly outperform SLPA [17] with different values of μ and $O_{m}$. In most cases, the performances of the NMLPA methods, especially NMLPA_Attr, are several times better than SLPA [17]. Furthermore, compared to NMLPA_NonAttr, NMLPA_Attr achieves more stable performances with the value of $O_{m}$ increasing, which indicates that our method is capable of making full use of the node attribute information of attributed networks.

### 5.2. Evaluation on Real-World Networks

We also evaluated the performance of NMLPA on real-world networks with overlapping communities. We used three attributed networks with ground-truth communities: Facebook [23], Flickr [21] and Twitter [24]. The network statistics are reported in Table 1.

Among these three networks, Facebook and Twitter are ego-networks, which are available in the Stanford Large Network Dataset Collection (https://snap.stanford.edu/data/). The ground-truth communities in Facebook are defined as social circles, while they are “lists” in Twitter. In Facebook, we regarded user profiles as node attributes. In Twitter, node attributes come from hashtags used by the user. To experiment on large networks, we used the combined networks of multiple ego-networks as the final datasets, which include 10 Facebook ego-networks and 973 Twitter ego-networks respectively.

Flickr is an image sharing network in which nodes represent users and edges represent follow relationships between users. Tags that users added to uploaded images are used as node attributes. In this network, we regarded the Flickr user groups as the ground-truth communities.

We compared NMLPA with four state-of-the-art community detection methods: SLPA [17], CESNA [27], SCI [22] and CDE [23]. All these methods consider both network structure and node attributes except for SLPA [17]. For all these baseline algorithms, we used the implementations provided by their authors and set their parameters by default. We used F1-score and Jaccard Similarity as our evaluation metrics, the same as the metrics used in synthetic network experiments.

Table 2 shows the community detection results of our method NMLPA and the four baseline methods on the three datasets mentioned above. The results indicate that NMLPA outperforms all baseline methods on all datasets for the overlapping community detection task. Specially, compared to SLPA [17], NMLPA achieves great promotion in the performance of community detection, which suggests the node-similarity based method in NMLPA can make full use of node attribute information. Since SCI [22] and CDE [23] are not able to deal with networks over 50,000 nodes in a reasonable time, we ignored the results of these two baseline methods on the Twitter network.

### 5.3. Parameter Sensitivity Analysis

To evaluate how the parameters of NMLPA affect its performance on community detection tasks, we conducted multiple experiments on the Facebook dataset with the parameter changing. The iteration number *t* and the label weight threshold *r* are two main parameters in our algorithm. In the interest of brevity, we fixed one parameter and then varied the value of another parameter to determine its impact on the community detection task.

**Figure 7:**
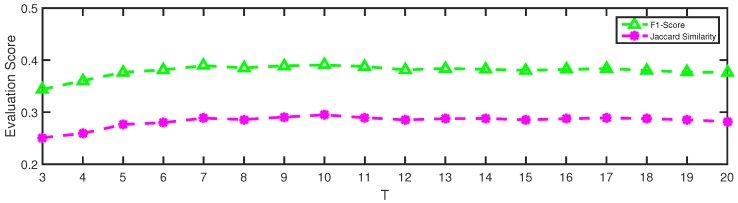
Varying the iteration number *t* from 3 to 20 with r=3 on the Facebook dataset.

Figure 7 shows the effect of performance (in terms of F1-score and Jaccard Similarity) when increasing the iteration number of our algorithm. In this case, we removed the evaluation results when t<3 since too few iterations hardly reflect the effect of our method on the results. It is clearly seen that the performance of NMLPA first goes up and then gradually becomes stable as the iteration number increases. This is mainly because the label propagation process of our method collects more and more useful information as the iteration number increases when this value is not too large. However, as the iteration number keep increasing, the distribution of node labels of the whole network gradually gets stable, which results in the stable performance when the value of *t* is large enough. It is worth noting that NMLPA achieves maximum scores with F1-score equaling 0.3905 and Jaccard similarity equaling 0.2947 when t=10, which is the same setting of our experiments on the Facebook dataset.

**Figure 8:**
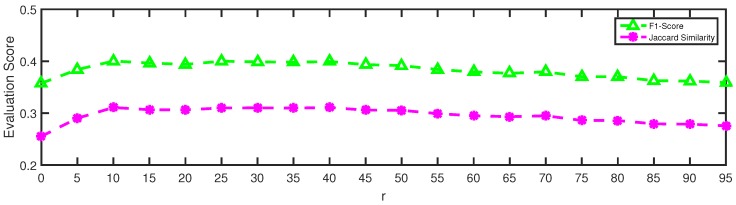
Varying the label weight threshold *r* from 0 to 95 with t=10 on the Facebook dataset.

In terms of the label weight threshold *r*, we present the results of varying *r* in Figure 8. At the beginning, the increase of *r* greatly improves the performance of NMLPA, which indicates that the pruning operation of our algorithm significantly helps to filter useless labels and thus NMLPA can find more accurate communities. However, as the value of *r* keeps increasing, the performance of NMLPA declines slowly. The reason behind this phenomenon is obvious since too large *r* removes redundant labels that may be useful for identifying community memberships. NMLPA achieves best performance with F1-score equaling 0.4003 and Jaccard similarity equaling 0.3109 when r=10, which is very close to our experimental results on the Facebook dataset.

### 5.4. Analysis of Detected Communities

We further analyzed the overlapping communities detected by our method. We chose the DELICIOUS dataset (http://ir.ii.uam.es/hetrec2011/datasets.html) from a social bookmarking system called *Delicious* whose 1867 users are interconnected in a social network generated form *Delicious* “mutual fan” relations. An attributed network was constructed by extracting 10,000-dimensional most popular bookmarks as the attributes of each user. Since the ground-truth communities of this network is unknown, the Louvain method [42] was used to set the number of communities to be 94. We then uncovered overlapping communities of this network using our method NMLPA.

**Figure 9:**
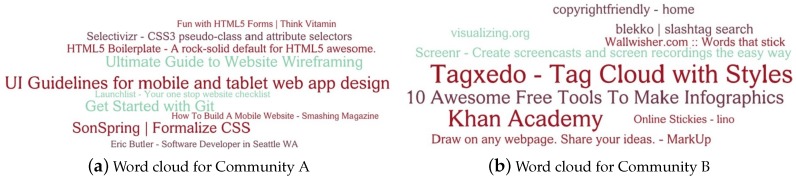
Word clouds for different communities detected by NMLPA. Top ten attributes of these communities are displayed here. The size of the word is proportional to its frequency in corresponding community.

Figure 9 shows the word clouds of two communities (denoted by Community A and Community B) detected by our method. For each detected community, we select the titles of ten most popular bookmarks, that is, the node attributes with most frequency in the corresponding community. The size of a word is proportional to its frequency in the corresponding community. Based on the content of the word cloud in Figure 9a, it is obvious that Community A is a group of programmers whose major work is about User Interface (UI) design. For example, the title “UI Guidelines for mobile and tablet web app design”, which is the largest title of this figure, tells us the topic of this community is about UI design. Specially, *HTML5* and *CSS* are the core components in UI design. The most widely used tool in source-code managements called *Git* also appears here. The topic of Community B in Figure 9b is probably about the multimedia production. For example, *Tagxedo*, the key word of the largest title “Tagxedo - Tag Cloud with Styles”, is a popular website that can turn words into a visually stunning word cloud. *Screenr* is a screen capture and annotation tool for web professionals. Besides, some multimedia search tools including *copyrightfriendly* and *blekko* are also frequent words in this community. In summary, the node attributes, i.e., the titles of bookmarks, are closely related in the same community, which indicates that the nodes in the same community detected by NMLPA are densely connected in terms of the semantic information.

## 6. Conclusions and Future Work

We propose NMLPA, a novel approach for detecting overlapping communities in attributed networks. By transforming the node attribute information into the weights of edges, our method is capable of combining the node attributes with the network topology while performing multi-label propagation on the whole network. We also use a pruning operation to control the number of labels in the multi-label propagation process. The communities selected after the post-processing process are regarded as the final results of our method. The extensive experiments on both synthetic networks and real-world networks indicate our method significantly outperforms several state-of-the-art community detection methods and is able to uncover good community structures in terms of the semantic information of nodes.

In the future, we would like to extend our method on multilayer networks in which nodes belong to the same set but edges carry different semantics. As a possible solution to this problem, merging multilayer networks into one network can make our method apply to multilayer networks conveniently. However, it is still a huge challenge to design an effective merging approach. Furthermore, we would like to develop our studies further on other complex networks such as weighted networks and heterogeneous networks.

## Figures and Tables

**Table 1:** Dataset Statistics. *n*, the number of nodes; *m*, the number of edges; *s*, the number of node attributes; *k*, the number of communities; AD, the average degree; AS, the average size of communities; AN, the average community memberships. We treated all these datasets as directed networks.

Dataset	*n*	*m*	*s*	*k*	AD	AS	AN
Facebook	4039	176,468	10	193	43.69	21.93	1.05
Flickr	16,710	1,432,126	1156	5436	85.70	273.10	88.84
Twitter	81,306	2,420,766	50	4065	29.77	12.51	0.63

**Table 2:** Performance comparison (in terms of F1-score and Jaccard Similarity) on attributed networks with overlapping ground-truth communities (bold numbers represent the best results). NA means that the task is not completed in limited time.

Dataset	F1-Score					Jaccard Similarity				
	SLPA	CESNA	SCI	CDE	NMLPA	SLPA	CESNA	SCI	CDE	NMLPA
Facebook	0.3058	0.3233	0.0854	0.3506	**0.3905**	0.2311	0.2244	0.0462	0.2408	**0.2947**
Flickr	0.0237	0.1041	0.0532	0.1324	**0.1444**	0.0122	0.0559	0.0246	0.0722	**0.0804**
Twitter	0.1028	0.2223	NA	NA	**0.2370**	0.0667	0.1374	NA	NA	**0.1542**
